# Supplementary material for: Bacterial cellulose spheroids as building blocks for 3D and patterned living materials and for regeneration
Source: Nat Commun. 2021 Aug 19;12:5027. doi: 10.1038/s41467-021-25350-8 (PMC8377073; doi:10.1038/s41467-021-25350-8)
Supplement: Supplementary file 1 — Supplementary Information [file 41467_2021_25350_MOESM1_ESM.pdf]

Supplementary Information

Bacterial cellulose spheroids as building blocks for 3D and patterned living materials and for regeneration

Joaquin Caro-Astorga[1,2], Kenneth T. Walker[1,2], Natalia Herrera Vargas[3], Koon-Yang Lee[3], Tom Ellis[1,2]*.

[1] Department of Bioengineering, Imperial College London, London, SW7 2AZ, UK,

[2] Imperial College Centre for Synthetic Biology, Imperial College London, London, SW7 2AZ, UK,

[3] Department of Aeronautics, Imperial College London, London, SW7 2AZ, UK,

* To whom correspondence may be addressed. Email: t.ellis@imperial.ac.uk

Supplementary Table 1. Culture condition variables tested to find the determinant parameters for BC spheroids production. Not all combinations of parameters were tested. Only the container and the OD600 of inoculation bacteria at the start of the culturing were found to have a critical effect.

Variable	Parameters
Inoculum age	1-10 days
Bacterial sub-culturing	1-4 subcultures
Container	14, 15, 50 ml plastic tubes; glass flasks of 100, 250, 500 and 1000 ml with plain and irregular wall; glass bottles of 100, 250, 500 and 1000 ml
Temperature	30°C and 25°C
Medium : air ratio in container	1:3, 1:4, 1:5, 1:10
Shaking conditions	150, 250 and 350 rpm in incubator; 100 rpm with magnet stirrer
Culture medium	HS, 2x HS, +1% ethanol, + 8% glycerol, 1% fructose
Time between seeding and shaking	0, 30 min, 1 hour and 2 hours
Inclination of tubes	0, 15, 45, 65 and 90 degrees
Preculture dilution	1:10, 1:100, 1:1000
Optical density of the final culture	0.1, 0.01, 0.001, 0.0001

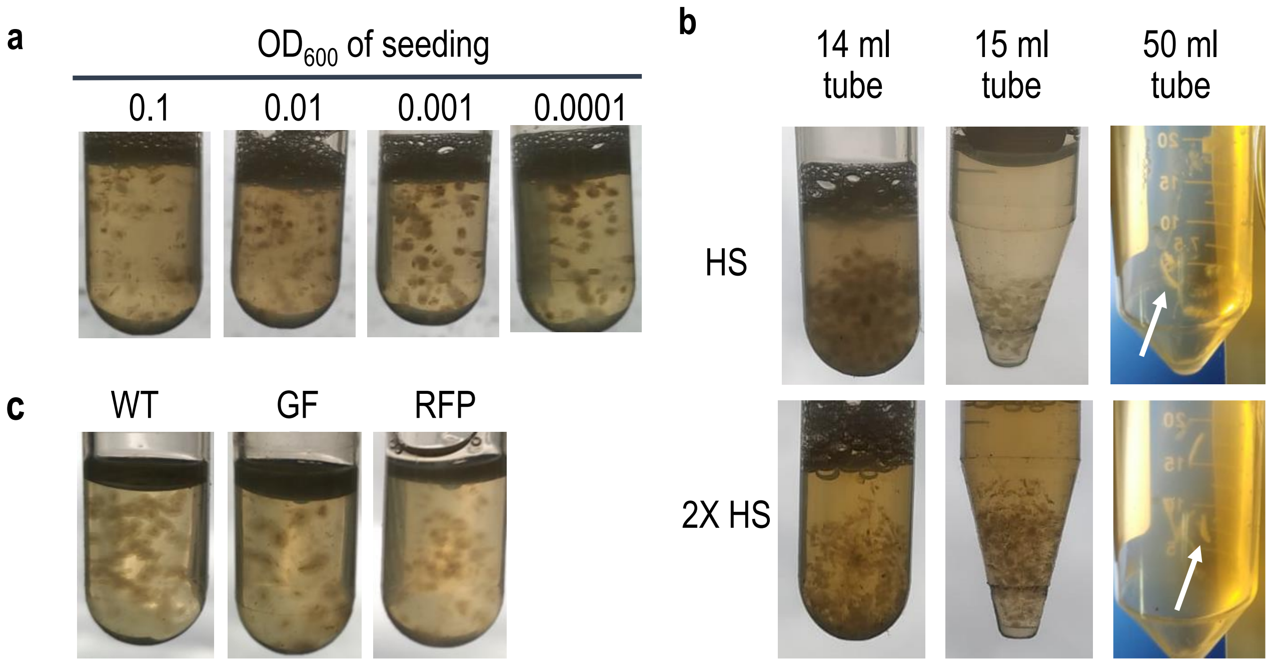

Supplementary Figure 1. BC spheroids growth conditions. a) Effect of Optical Density of the culture. Only OD below 0.001 produce a high yield and good quality rounded spheroids. Higher densities produce fibrous material and interconnected clumps. b) Effect of the shape of the tube and media concentration on spheroids production. Production of BC spheroids in 50 ml tubes was unsuccessful, with 14 ml culture tubes being the preferred choice. 2x HS medium produce higher quantities of spheroids, but they generally of small size that do not grow with more time of incubation. c) Cells of *K. rhaeticus* transformed with plasmids encoding high expression of superfolder GFP (centre) or RFP (right) produced similar spheroids to those made by the wild type (WT) strain (left).

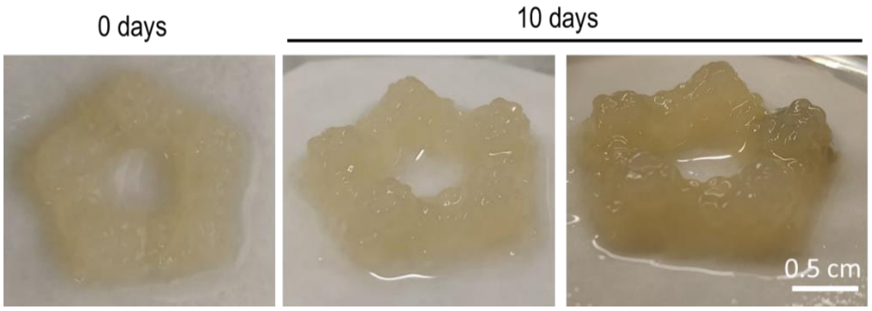

Supplementary Figure 2. BC spheroids as building blocks. Growth of a pentagonal 3D shape with elevated apexes constructed using BC spheroids. Spheroid seeding (left) and result after 10 days of growth (right).

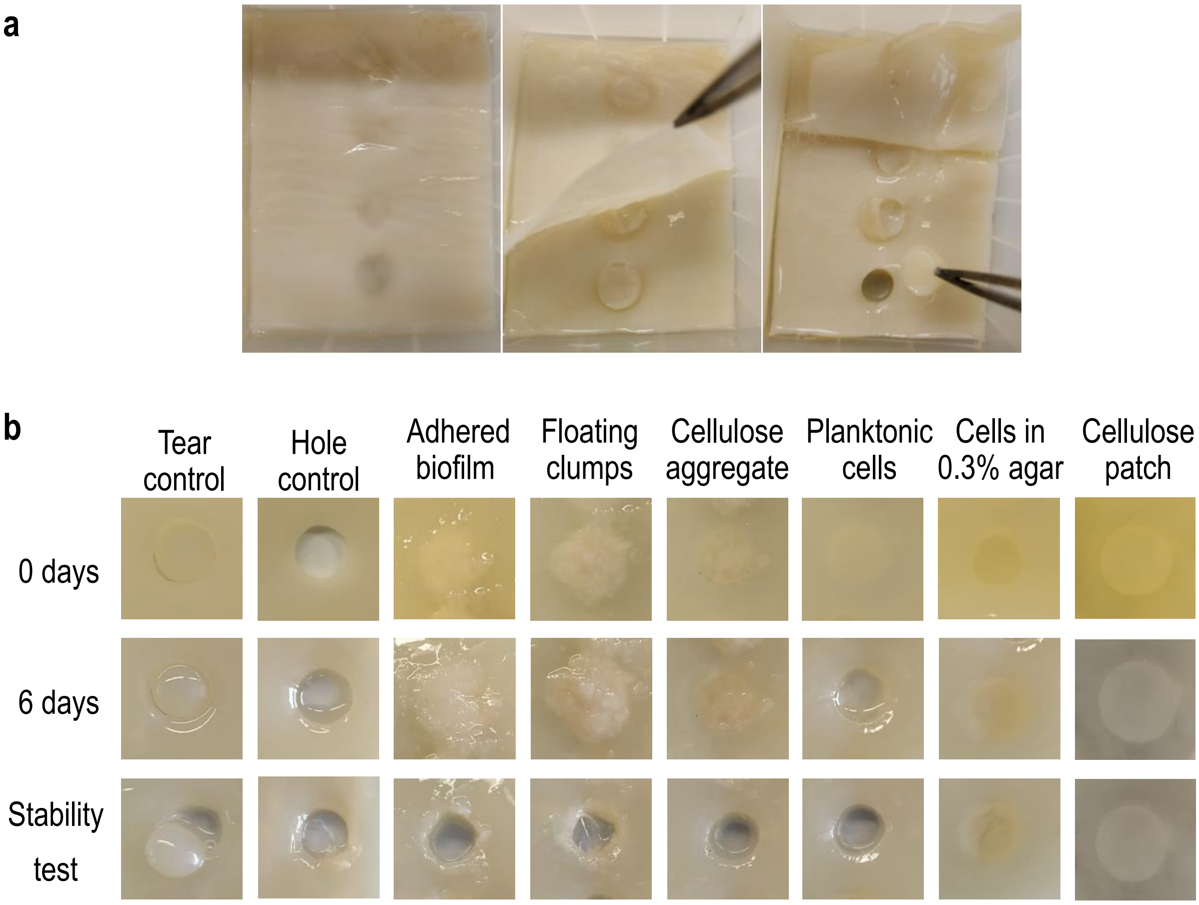

Supplementary Figure 3. BC pellicle regeneration. a) Repair assay of a bacterial cellulose pellicle damaged with a hole puncture then incubated with static aeration for 7 days at 30°C in fresh HS media supplemented with 2% glucose. Over the original BC, a new pellicle grows that is poorly attached to the original pellicle and can be removed easily with forceps. b) Repair assay of a bacterial cellulose pellicle damaged with a hole puncture. Images shown are representative of three replicates. Upper line shows images of seeding the repairing bacteria. Middle and bottom line shows images after 6 days of incubation and after the stability test, respectively. Cells in different physiological state were used to heal the damage and incubated for 6 days at 30°C. The source of cells were: i) fragments of biofilm adhered to the wall of a flask after 4 days in shaking conditions; ii) floating clumps formed in shaking conditions from an initial culture set with high cell density (OD_{600} ~0.5); iii) cellulose aggregates present in the culture medium under the pellicle formed in static culture; iv) a pellet of cells grown in shaking conditions with cellulase, centrifuged, washed with HS and centrifuged again; v) cells from (iv) embedded in a 0.3% HS agar matrix at 40°C and immediately placed in the pellicle before it solidifies; vi) a cellulose patch of slightly bigger dimensions than the hole produced in the pellicle, used to force the edges of the patch and the hole to be in close contact; vii) cellulose spheroids from a day 3 shaking culture. Although agar driven repair and cellulose patches passed the stability test, the BC material has not actually regrown.

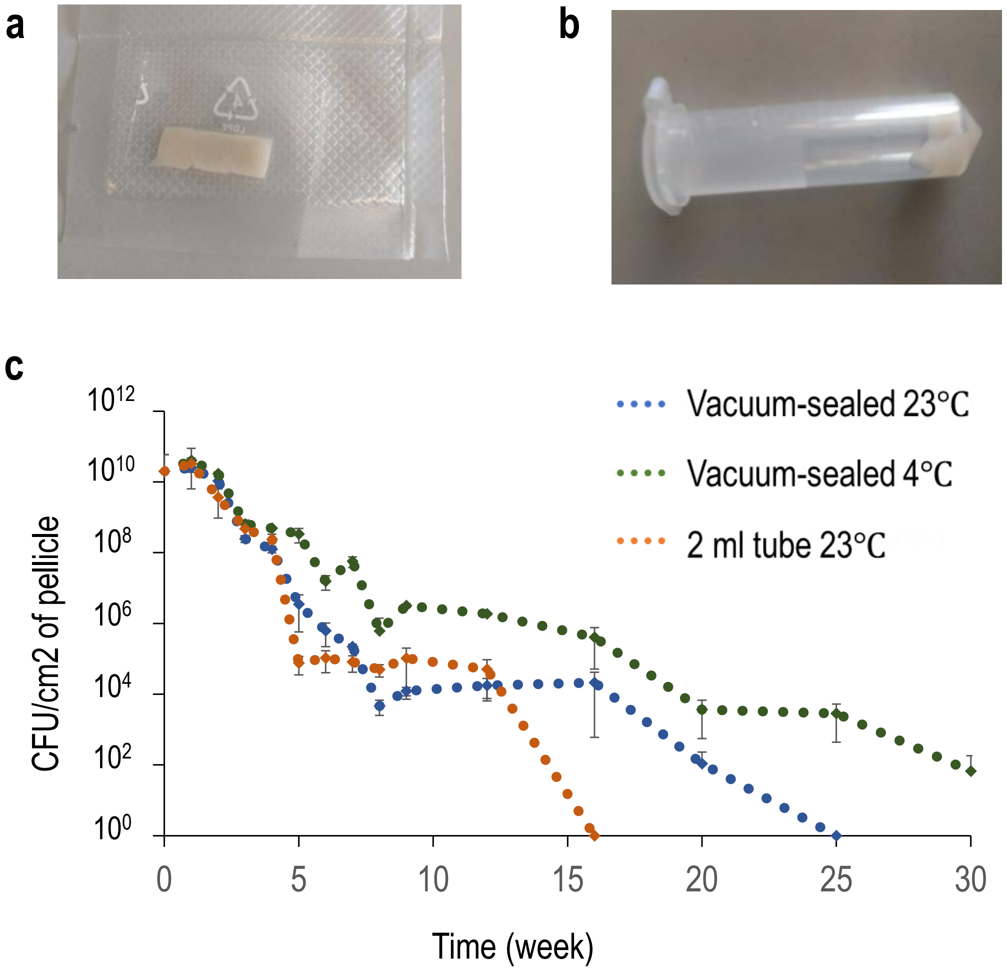

Supplementary Figure 4. BC cell survival. a) Sealed bag containing 3 squared pellicles. b) Three pellicles stored in a 2-ml tube as a non-sealed container. c) Graph of *K. rhaeticus* cell survival over time within BC pellicles stored in vacuum-sealed plastic bags or in 2-ml tubes at 4°C and 23°C. Plotted values are the mean of 3 biological replicates of 4 technical replicates each. Error bars indicate SD. Source data are provided as a Source Data file.

a

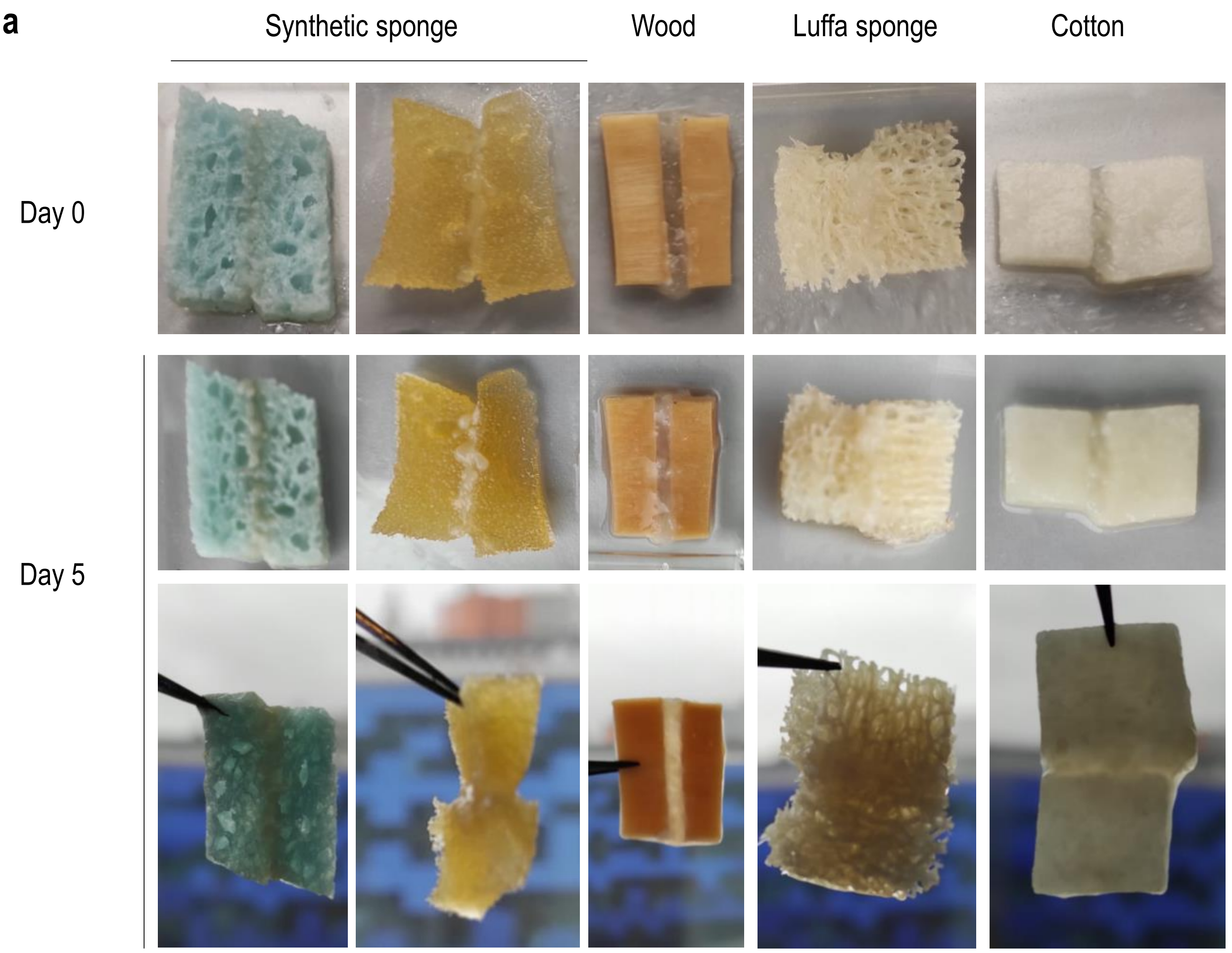

b

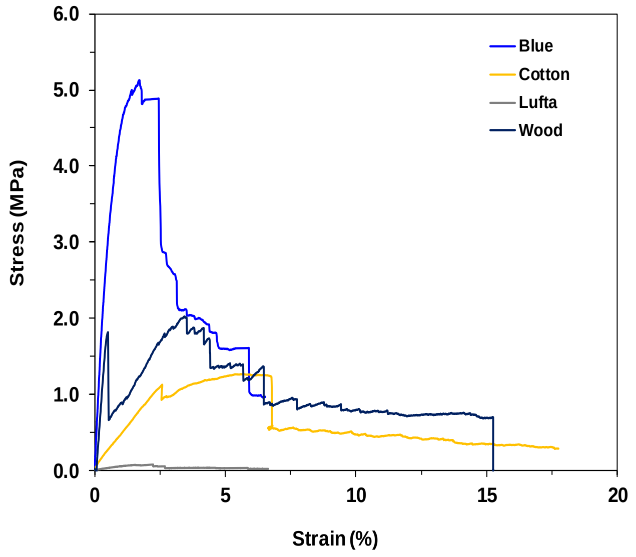

	Modulus (GPa)	Max. σ (MPa)	Max. ε (%)	Max. Force (N)
Blue sponge	0.7	5.13	6.53	5.02
Cotton	0.1	1.42	15.14	2.70
Luffa	0.01	0.09	6.94	5.72
Wood	0.5	2.03	15.25	1.08

Supplementary Figure 5. BC spheroids for materials integration. a) Images of fused pieces of synthetic sponges, cotton and wood using BC spheroids at Day 0 (top row), and after 5 days of incubation at 30°C (middle row) and when lifted with forceps (bottom row). b) Mechanical test of fused pellicles after 5 days of incubation on each side of the fusion BC spheroids-material to improve integration. Source data are provided as a Source Data file.

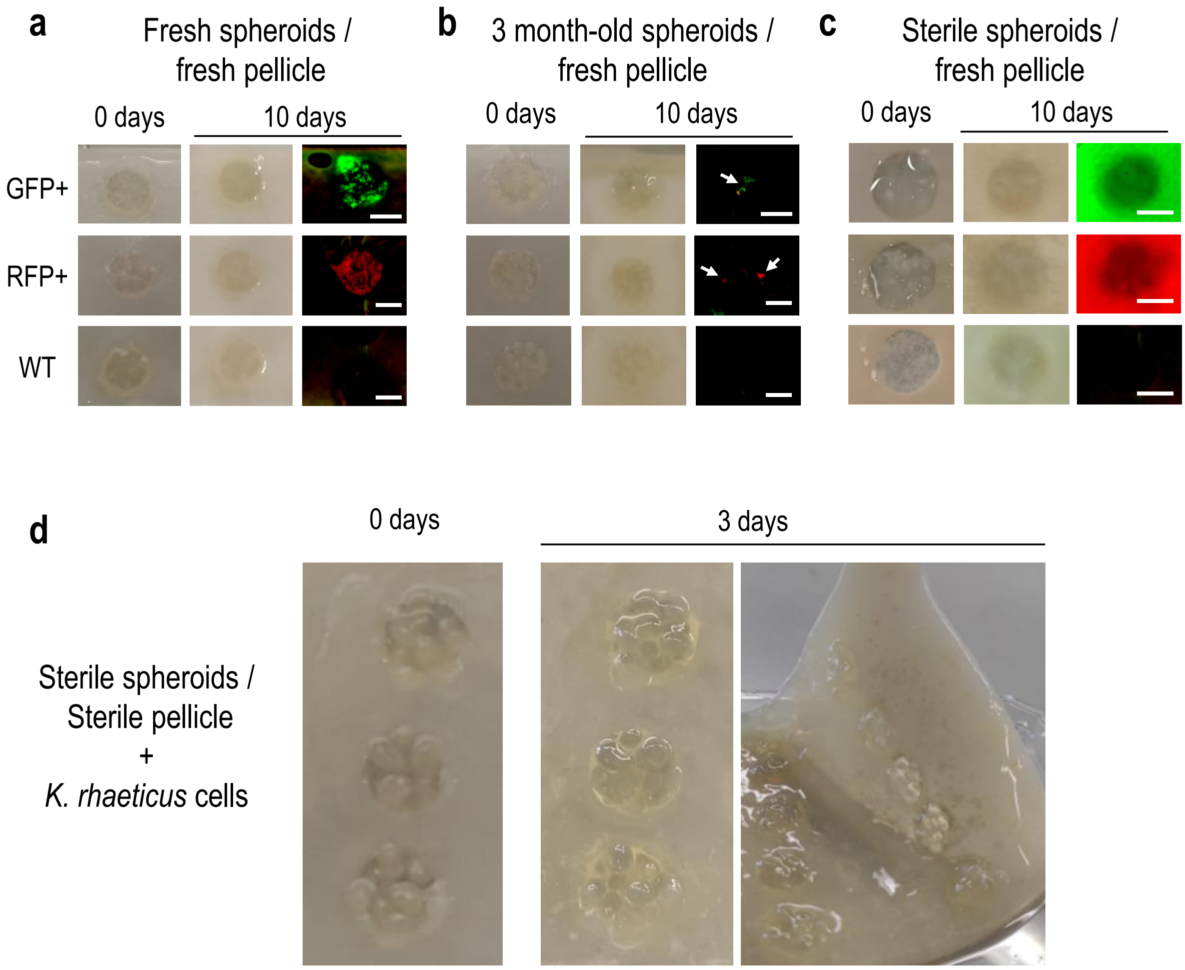

Supplementary Figure 6. BC pellicle regeneration. a) Regeneration of a fresh BC pellicle using fresh BC spheroids from wildtype (WT), and GFP- and RFP-expressing *K. rhaeticus* (left: visible channel at day of seeding, middle: visible channel after 10 days incubation, right: fluorescence channel after 10 days). b) Result of the regeneration of a fresh BC pellicle using 3-month old BC spheroids from wildtype (WT), and GFP- and RFP-expressing *K. rhaeticus* (left: visible channel at day of seeding, middle: visible channel after 10 days incubation, right: fluorescence channel after 10 days). Arrows indicate small regions of fluorescence. c) Result of the regeneration of fresh BC pellicles using sterile purified BC spheroids. Fresh pellicles were grown from wildtype (WT), and GFP- and RFP-expressing *K. rhaeticus* (left: visible channel at day of seeding, middle: visible channel after 10 days incubation, right: fluorescence channel after 10 days). Bars equal 5 mm. d) Reparation of a purified BC pellicle using purified spheroids and adding 100 µl of a cell suspension of OD600 = 1 and 3 days of incubation at 30°C.

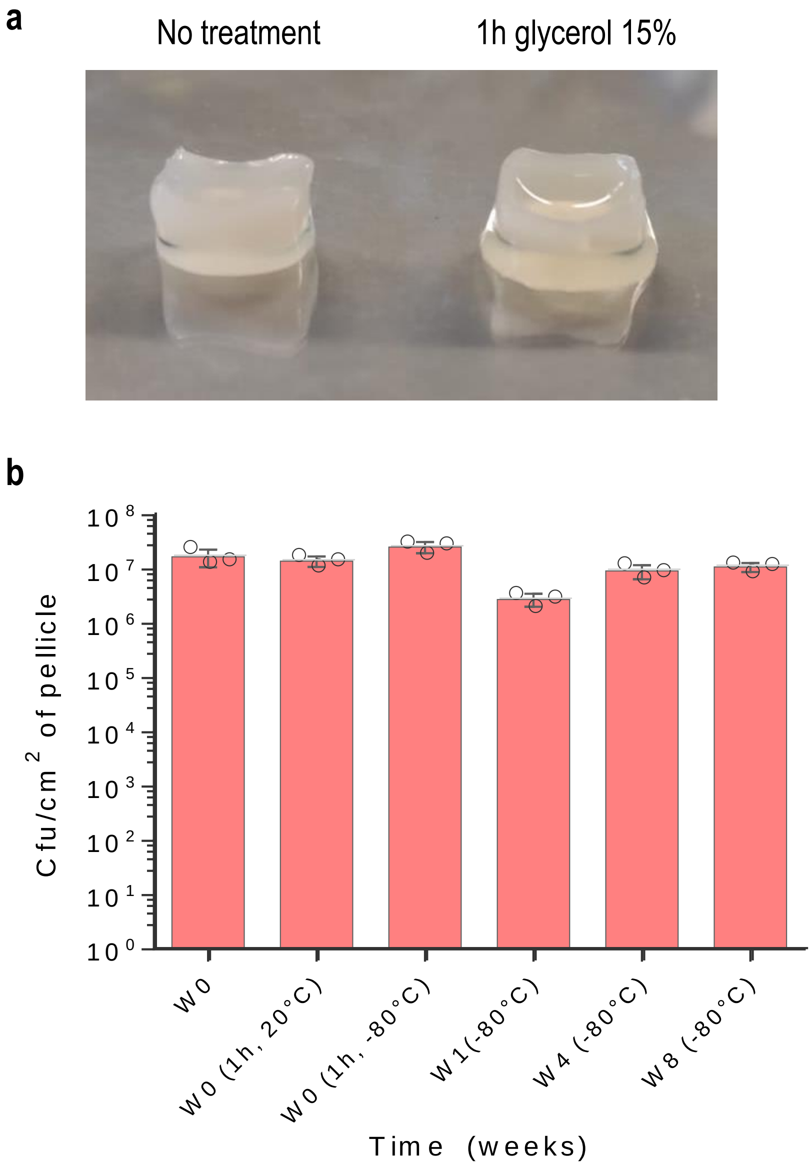

Supplementary Figure 7. BC cell conservation within the pellicle material at -80°C with and without 15% glycerol addition. a) Representative image of BC pellicle material grown in deep weel 96 well plates, either not treated (left) or treated by soaking in 15% glycerol for 1 hour (right). b) Graph of *K. rhaeticus* cell survival over time within BC pellicle samples treated by soaking in 15% glycerol for 1 h, and then stored at -80°C for 1 hour (1h), 1 week (1w) and 1 month (1m). A control of cell count from a fresh pellicle sample is shown (0h). Plotted values are the mean of 3 biological replicates of 4 technical replicates each. Error bars indicate standard deviation

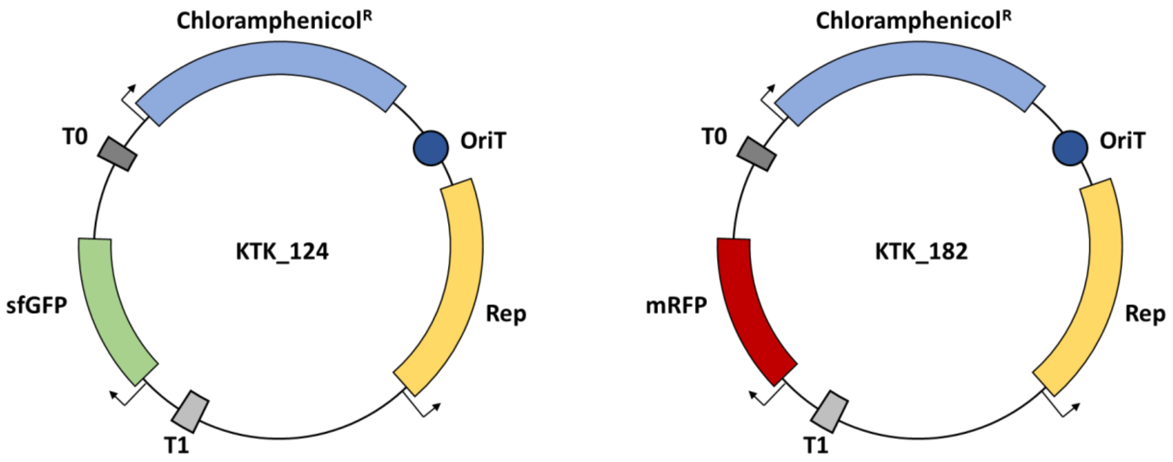

Supplementary Figure 8. Plasmids maps. Schematic of the genetic constructs of the plasmids used to functionalize bacteria to produce fluorescent bacterial cellulose spheroids.
