# Supplementary material for: Bacterial cellulose spheroids as building blocks for 3D and patterned living materials and for regeneration
Source: Nat Commun. 2021 Aug 19;12:5027. doi: 10.1038/s41467-021-25350-8 (PMC8377073; doi:10.1038/s41467-021-25350-8)
Supplement: Supplementary file 8 — Description of Additional Supplementary Files [file 41467_2021_25350_MOESM8_ESM.pdf]

Title: Supplementary Movie 1.
Description: Early stages of the entangling process of prespheroids formation. Time-lapse of *K. rhaeticus* cells growth for 2 hours in microfluidic plate with HS media supplemented with Fluorescence Brightner 28 to stain cellulose bands (blue). Bands of bacterial cellulose are produced continuously while bacteria continue the cell division process, resulting in branched cellulose bands. In the top area of the plate, a globular tangle of bacteria producing bacterial cellulose bands is forming the first stages of a spheroid.

Title: Supplementary Movie 2.
Description: BC spheroids as building blocks. Pentagonal 3D shape built using BC spheroids on filter paper. After seeding the spheroids, the piece was incubated for 10 days at 30°C. The video shows how the spheroids regrow the surface in the 3 D shape designed and show its flexibility and its consistency.

Title: Supplementary Movie 3.
Description: BC spheroids for materials integration. Video of fused pieces of synthetic and natural sponges, cotton and wood using BC cellulose spheroids at seeding time and after 5 days of incubation at 30°C.

Title: Supplementary Movie 4.
Description: BC pellicle repair. Video of a purified (sterile) BC pellicle repaired using purified (sterile) BC spheroids and the addition of *K. rhaeticus* cells grown in shaking liquid culture (with cellulase added). The pellicle with spheroids was incubated for 5 days at 30°C for repair.
